# Modeling Anisotropic Electrical Conductivity of Blood: Translating Microscale Effects of Red Blood Cell Motion into a Macroscale Property of Blood

**DOI:** 10.3390/bioengineering11020147

**Published:** 2024-02-01

**Authors:** Alireza Jafarinia, Vahid Badeli, Thomas Krispel, Gian Marco Melito, Günter Brenn, Alice Reinbacher-Köstinger, Manfred Kaltenbacher, Thomas Hochrainer

**Affiliations:** 1 Institue of Strength of Materials, Graz University of Technology, Kopernikusgasse 24/I, 8010 Graz, Austria; thomas.krispel@tugraz.at (T.K.); hochrainer@tugraz.at (T.H.); 2 Institute of Fundamentals and Theory in Electrical Engineering, Graz University of Technology, Inffeldgasse 18, 8010 Graz, Austria; vahid.badeli@tugraz.at (V.B.); alice.koestinger@tugraz.at (A.R.-K.); manfred.kaltenbacher@tugraz.at (M.K.); 3 Institute of Mechanics, Graz University of Technology, Kopernikusgasse 24/IV, 8010 Graz, Austria; gmelito@tugraz.at (G.M.M.); 4 Institute of Fluid Mechanics and Heat Transfer, Graz University of Technology, 8010 Graz, Austria; guenter.brenn@tugraz.at (G.B.)

**Keywords:** anisotropic electrical conductivity, electrical conductivity of blood, computational fluid dynamics, bioimpedance signals, impedance cardiography, impedance plethysmography

## Abstract

Cardiovascular diseases are a leading global cause of mortality. The current standard diagnostic methods, such as imaging and invasive procedures, are relatively expensive and partly connected with risks to the patient. Bioimpedance measurements hold the promise to offer rapid, safe, and low-cost alternative diagnostic methods. In the realm of cardiovascular diseases, bioimpedance methods rely on the changing electrical conductivity of blood, which depends on the local hemodynamics. However, the exact dependence of blood conductivity on the hemodynamic parameters is not yet fully understood, and the existing models for this dependence are limited to rather academic flow fields in straight pipes or channels. In this work, we suggest two closely connected anisotropic electrical conductivity models for blood in general three-dimensional flows, which consider the orientation and alignment of red blood cells (RBCs) in shear flows. In shear flows, RBCs adopt preferred orientations through a rotation of their membrane known as tank-treading motion. The two models are built on two different assumptions as to which hemodynamic characteristic determines the preferred orientation. The models are evaluated in two example simulations of blood flow. In a straight rigid vessel, the models coincide and are in accordance with experimental observations. In a simplified aorta geometry, the models yield different results. These differences are analyzed quantitatively, but a validation of the models with experiments is yet outstanding.

## 1. Introduction

The early detection of cardiovascular diseases (CVDs) is an important healthcare objective, given that they are the leading global cause of mortality. While conventional diagnostic methods, such as CT or MRI scans and invasive procedures, have been effective, they are costly and potentially risky for patients [1,2]. There is a need for a rapid, non-invasive, and cost-effective alternative to enable the early detection and personalized clinical decision-making for CVDs. Bioimpedance signals promise a compelling solution with these advantages. This study introduces an innovative modeling approach for the electrical conductivity of blood to enable the investigation of bioimpedance measurements.

Bioimpedance signals, including impedance plethysmography (IPG) and impedance cardiography (ICG) signals, offer insights into the physiological and pathophysiological processes within blood vessels. Unlocking the diagnostic potential of bioimpedance signals depends on the ability to classify these signals [3]. The quantitative interpretation of bioimpedance signals presents a significant challenge, and this field remains largely unexplored, explaining its absence in current clinical practices. This challenge becomes more apparent when addressing CVDs characterized by altered local hemodynamics and blood flow disruptions, such as stenosis, aneurysms, aortic dissection, atherosclerosis, and thrombosis.

In clinical practice, bioimpedance signals are obtained by injecting a low-frequency electric current into the body. Since blood is a highly conductive material when compared with the surrounding tissues and organs (lungs, bones, muscles, etc.), the electric current travels preferentially through the blood vessels. Therefore, bioimpedance signals are highly sensitive to changes in the electrical conductivity of blood [4,5,6]. The electrical current primarily flows through the blood plasma, as red blood cells (RBCs) are electrically non-conductive at the frequencies relevant to impedance measurements, i.e., up to electrical frequencies of 1 MHz [7,8]. Because of their non-conductive nature, RBC concentration and complex motions significantly influence the electrical conductivity of blood. A higher concentration of RBCs results in an increased presence of non-conductive material within the blood, reducing the overall conductivity. When red blood cells form channel-like pathways within blood vessels, it facilitates an easier flow of electrical current through the conductive plasma, increasing the overall conductivity.

Local hemodynamics affect the RBCs motion, particularly their orientation, alignment, and deformation, which depend on the state of shear rate and shear stress. Given that RBCs are non-conductive and non-spherical, their orientation, alignment, and deformation affect the anisotropic electrical conductivity of blood [7,9,10]. Consequently, RBC motion plays a pivotal role in the analysis of bioimpedance signals linked to CVDs.

The study of the electrical conductivity of blood was started by Maxwell [11], Rayleigh [12], and Fricke [13]. The first two initially developed a basic theory for determining the size and shape of spherical particles in a suspended fluid. Successively, Fricke [13] extended their work to consider the electrical conductivity of randomly distributed ellipsoidal particles in a fluid. In the literature, this theory is known as the Maxwell–Fricke theory, and it allows for the computation of the electrical conductivity of stationary blood based on the blood temperature, volume fraction, and the shape of RBCs. Many years later, Edgerton [9] extended the Maxwell–Fricke theory by including the probability distribution of RBCs orientation in the blood flow and showing that their distribution is a function of shear rate. Ref. Visser [14] confirmed the result of Edgerton and also indicated that the orientation of RBCs is the main cause of blood conductivity changes.

**Figure 1:**
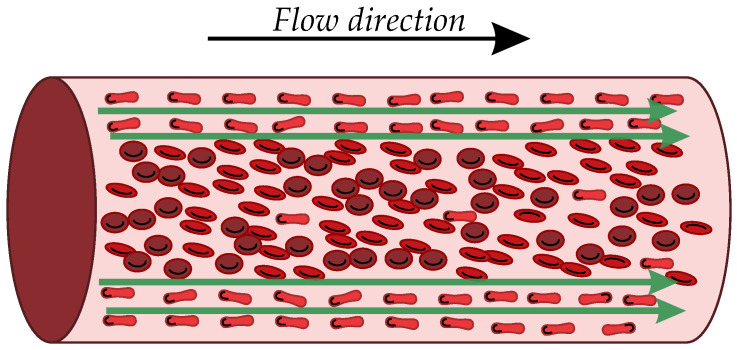
An idealized schematic of how RBCs align in the flow direction, creating channel-like paths near the vessel wall. The green arrows indicate the passage of the electrical current.

Recently, Hoetink et al. [7] developed a model based on the Maxwell–Fricke theory for computing the blood conductivity of a diluted suspension of ellipsoidal particles, which simulates blood in a steady flow in a rigid vessel. Considering RBCs as ellipsoidal particles, their results showed that, due to high values of shear stress, the RBCs deform and orient such that one of their long axes is parallel to the streamlines of the blood flow. This new configuration causes a substantial change in the electric current path through the blood [7,8]. In particular, the electrical conductivity of blood in the direction of flow increases due to channel-like paths available between the aligned RBCs. Channel-like paths are shown in Figure 1.

Gaw et al. [10] extended the investigation and reported the effects of pulsatile blood flow on electrical conductivity in a rigid vessel. Theoretically and experimentally, it was shown that, when the velocity increases during systole, there is a robust linear relationship between the average velocity and the conductivity of the blood. Similarly, a decrease in impedance is observed when blood velocity decreases, i.e., during diastole.

The recent modeling approaches developed by Hoetink et al. [7] and Gaw et al. [10] inspired several application studies for the simulation of bioimpedance signals [15,16], in particular in the field of aortic dissection [3,4]. However, these approaches focused on the analytical and numerical solutions for one-dimensional (1D) computations of the electrical conductivity of blood, and none offered the possibility to model and compute the 3D anisotropic electrical conductivity of blood as a field variable. For example, a previous study by Badeli et al. [4] showed the important application of the ICG method in detecting aortic dissection. However, the isotropic assumption imposed by the 1D formulation led to ignored changes in the blood conductivity values due to varying flow direction, local flow hemodynamics, and disturbances due to pathology.

An initial attempt toward a more accurate description of the conductivity field was performed by Jafarinia et al. [17] and Badeli [18]. A two-dimensional (2D) model for computing anisotropic conductivity as a time-dependent field variable was developed. The studies initiated the use of computational fluid dynamics (CFD) simulations for the computation of blood flow conductivity. Through a multiphysics approach, combining the electromagnetic and CFD simulations, the authors showed that, by tracking ICG signals, it is feasible to specify the changes in false lumen thrombosis in the case of aortic dissection. Recently, Badeli et al. [19] extended the 2D conductivity model to be used in three-dimensional (3D) multiphysics CFD simulations with some simplifications, including the assumption that the RBCs are prolate spheroid. As a result, the simulated bioimpedance signals confirmed that physiological changes, such as thrombosis, are trackable by monitoring the impedance changes. However, it was noted that the simplifications had caused some inaccuracies in the simulated bioimpedance signals.

The presented study aims to develop a 3D anisotropic electrical conductivity model capable of translating the microscopic effect of RBCs orientation and alignment into the macroscopic property of blood in a general 3D blood flow. The motion of RBCs is influenced by hemodynamic conditions, and the new model shall be based on experimental observations related to the orientation of RBCs in shear flows, which will be discussed in the next section. However, there are two alternative interpretations for what hemodynamic characteristic determines the orientation of the RBCs, which happen to coincide in the experimentally studied flow conditions. We therefore developed two 3D models based on either characteristic. These models share their root in the previous conductivity model proposed by Gaw et al. [10] and further studied by Melito et al. [20], which will likewise be introduced in Section 2. In Section 3, we validate the models in a pipe flow and compare their results to a flow in a simplified aorta geometry. The differences between the models are discussed in detail in Section 4, and we offer conclusions in Section 5.

## 2. Materials and Methods

Since the orientation and deformation of RBCs are the cause of anisotropic blood conductivity, it is essential to understand their complex motion in the blood flow. Several experimental studies have investigated the behavior of RBCs in Couette and Poiseuille flow fields, including Fischer et al. [21], Goldsmith et al. [22], Keller and Skalak [23], Bitbol [24], Schmid-Schönbein and Wells [25]. These studies suggest that RBCs exhibit two types of motion. First, unsteady motions like flipping, tumbling, and rolling, in which the biconcave shape of RBCs remains unchanged. Second, a steady motion where the RBCs undergo deformation into ellipsoidal particles due to high shear stress. In the steady motion, the RBCs maintain a steady orientation with their membrane circulating around the interior viscous fluid (cytoplasm) [26]. This motion is called tank-treading, where the rotating motion of the membrane transfers the tangential stresses of the flow to the cytoplasm. In fact, the cytoplasm recirculates and dissipates the energy transferred from the external flow, which allows the RBCs to keep a steady orientation and shape [26].

The two motions can occur simultaneously in a blood vessel. The probability of finding differently oriented RBCs varies depending on the shear rate and shear stress. The unsteady rigid-body-like motions are seen in low shear rates. There exists a gradual transition to tank-treading steady motion with increasing shear rate, where eventually the RBCs orient approximately in the direction of the flow [21,22,23]. According to Gaw et al. [10], the RBCs in unsteady motions are categorized as randomly oriented, while in the tank-treading motion, they are aligned in a preferred orientation determined by characteristics of the local blood flow.

Knowing the orientation of RBCs allows for computing the anisotropic blood conductivity in a fully aligned state. Therefore, in the following, the tank-treading motion is investigated in detail in order to extract the required information to determine the orientation of RBCs. Later, the computation of conductivity will be combined with the conductivity of blood with randomly oriented RBCs in order to treat different degrees of RBCs alignment.

**Figure 2:**
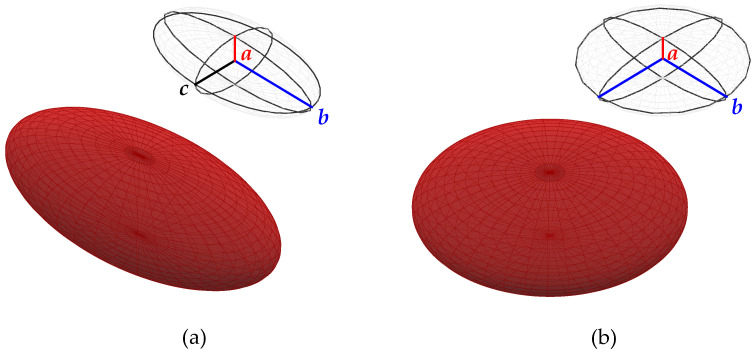
A triaxial ellipsoidal particle (**a**) characterized by a short (2a), an intermediate (2c), and a long (2b) axis and an oblate spheroid (**b**) with a short (2a) and two equal long axes (2b). See Table 1 for the values of *a* and *b*.

**Figure 3:**
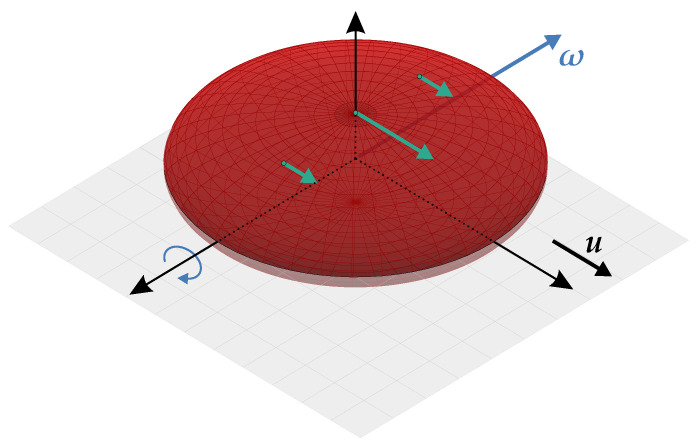
An idealized schematic of a tank-treading ellipsoidal RBC near a vessel wall with a high shear rate. The RBC is assumed to be an ellipsoidal particle with two equal long axes with the length 2b and one short axis of length 2a. The shear plane, shown in gray, is the plane of maximum shear stress containing the velocity vector u. The vorticity vector ω, is shown in a blue vector. The curved blue arrow indicates the cavity flow of the cytoplasm. The green arrows on the RBCs membrane indicate the local membrane speed due to the tank-treading motion.

The experiments of Fischer et al. [21] and Minetti et al. [27] showed that the RBCs in the tank-treading motion are triaxial ellipsoidal particles with a short (minor), an intermediate, and a long (major) axis, see Figure 2. According to their experiments, two primary conclusions are derived:

- The RBCs are oriented such that the intermediate and major axes are in a plane of maximum shear stress, which we shall call a ‘shear plane’ in the following. There exist two perpendicular shear planes because the viscous stress tensor τ is symmetric. The intermediate and major axes of the RBCs are found to lie in the shear plane, which mostly contains the flow direction. The major axis of ellipsoidal tank-treading RBCs is found to be parallel to the flow direction;
- The intermediate axis is parallel to the vorticity vector of the flow. Also, in this case, the major axis of the RBCs is parallel to the flow direction. Figure 3 shows an idealized schematic of a tank-treading RBC in shear flows.

As will be discussed in more detail in Section 4, in the case of the experimentally studied Couette and Poiseuille flows, it so happens that the shear plane, which contains the velocity vector, coincides with the plane spanned by the vorticity vector and the velocity vector. These planes do not coincide in general 3D flow fields and from the literature it is not clear whether shear stresses or the vorticity vector chiefly determine the orientation of tank treading RBCs.

Before introducing the models, we note that Edgerton [9], Bitbol [24], and Gaw et al. [10] advocated that for the computation of blood electrical conductivity in the cardiovascular system, it is reasonable to consider the RBCs as ellipsoidal particles with two equal long axes and one short axis, i.e., a symmetry axis, meaning that the RBCs are assumed to be oblate spheroids, see Figure 2b. We adopt this simplification in the current study. With this assumption, the orientation of RBCs is fully specified by the symmetry axis.

### 2.1. Modeling RBCs Motion

Considering the conclusions derived from the mentioned experiments and assuming RBCs to be oblate spheroids, the tank-treading RBCs are either considered to be oriented such that their flat surface (the surface spanned by the long axes) is parallel to the shear plane; such that the symmetry axis of RBCs is normal to the shear plane. Or such that the long axes of the RBCs are in the direction of flow and vorticity, suggesting that the symmetry axis of RBCs is normal to the plane spanned by the velocity and vorticity vectors. Figure 3 depicts these considerations at an idealized schematic of a tank-treading RBC with two long equal axes 2b and the symmetry axis 2a. The tank-treading motion of the RBC is indicated by the green arrows on the RBCs membrane. In Figure 3, the gray plane is the shear plane containing the velocity vector u, while the RBCs symmetry axis 2a is parallel to the normal of this shear plane. The long axes 2b are parallel to the shear plane, with one of them parallel to the velocity vector u and the other one to the vorticity vector ω.

Based on these observations, we propose the following two models to find the direction of the symmetry axis of RBCs and, consequently, their orientation in the tank-treading motion:

- ***The eigenvector model***: The direction of the symmetry axis is determined by the normal vector of the shear plane, which contains or mostly contains the velocity vector. The normal vector can be computed using the eigenvectors of the viscous stress tensor;
- ***The velocity–vorticity model***: the direction of the symmetry axis is determined by computing the cross product of vorticity and velocity, i.e., the so-called Lamb vector [28].

Up to now, our focus has been on tank-treading steady motion of RBCs occurring at high shear rates. However, in low shear rates, the orientation of RBCs is random [21]. There exists a gradual transition between complete randomness and full alignment in a steady orientation of RBCs, which is a function of shear rate. In principle, in the case of the blood flow in a vessel, the RBCs near the wall, where the shear rate is high, are assumed to be aligned to the flow direction [10]. They are randomly oriented in the middle of the vessel where the shear rate is zero [7,10]. For intermediate shear rates, Gaw et al. [10] assumed a gradual transition from random orientation in low shear rates and full alignment at high shear rates. In the current study we adopt this assumption together with the shear rate dependent transition function introduced by Gaw et al. [10], compare Equation (15) in Section 2.3, where we also specify what we consider as high, intermediate, and low shear rates.

Next, the computation of the direction of RBCs symmetry axis using eigenvector and velocity–vorticity models is explained. Knowing the orientation of RBCs, eventually, the electrical conductivity tensor of blood in a general three-dimensional (3D) flow is defined.

#### 2.1.1. Eigenvector Model

The global Cartesian coordinates system, which will be used in the numerical simulations of blood flow, is defined by *x*, *y*, and *z* axes, with the unit basis vectors $e_{x}$, $e_{y}$, and $e_{z}$. We assume that the viscous stress tensor τ has three distinct eigenvalues and define the orthonormal basis vectors $e_{1}$, $e_{2}$, and $e_{3}$ as the eigenvectors corresponding to maximum, intermediate, and minimum eigenvalue, respectively. These basis vectors form the viscous stress tensor’s principal coordinate system and are locally defined because τ is a field variable. The maximal shear stress in the system is given by the difference between the maximum and minimum eigenvalues of the stress tensor. The maximal shear stress is obtained as the magnitude of the in-plane component of the stress vector in surfaces with normal vectors along ‘the diagonal’ between the first and the third eigenvector. Note that since the directions of the eigenvectors are only defined up to a sign, there are two maximum shear planes with orthogonal normal vectors. These normal vectors may be obtained by rotating $e_{1}$ (or $e_{3}$) around the $e_{2}$-axis in positive or negative $45^{\circ }$. In the eigenvector model, we assume that the minor axis of the RBC $e_{\alpha }$ is aligned with the normal of the shear plane, which ideally contains the velocity vector (at high shear rates) or which contains the larger component of the velocity vector upon projection into the shear plane.

Equivalently, $e_{\alpha }$ (i.e., the sought shear plane normal) shall have a larger angle to the velocity vector u than the normal of the alternative shear plane. The two (up to a sign) $45^{\circ }$ rotations of the eigenvector $e_{1}$ around $e_{2}$, are the unit vectors $\frac{1}{\sqrt{2}}\left(e_{1}+e_{3}\right)$ and $\frac{1}{\sqrt{2}}\left(e_{1}-e_{3}\right)$. Accordingly, we define the direction of the short axis of the RBCs as follows:

(1) $$e_{\alpha }^{\mathrm{EV}}:=\left\{\begin{array}{cc} \frac{1}{\sqrt{2}}\left(e_{1}-e_{3}\right)\; & \mathrm{if}\;|\langle u,\frac{1}{\sqrt{2}}\left(e_{1}+e_{3}\right)\rangle \left|\;>\;\right|\langle u,\frac{1}{\sqrt{2}}\left(e_{1}-e_{3}\right)\rangle |\\ \frac{1}{\sqrt{2}}\left(e_{1}+e_{3}\right)\; & \mathrm{otherwise}, \end{array}\right.$$

where the superscript EV refers to the eigenvector model.

**Figure 4:**
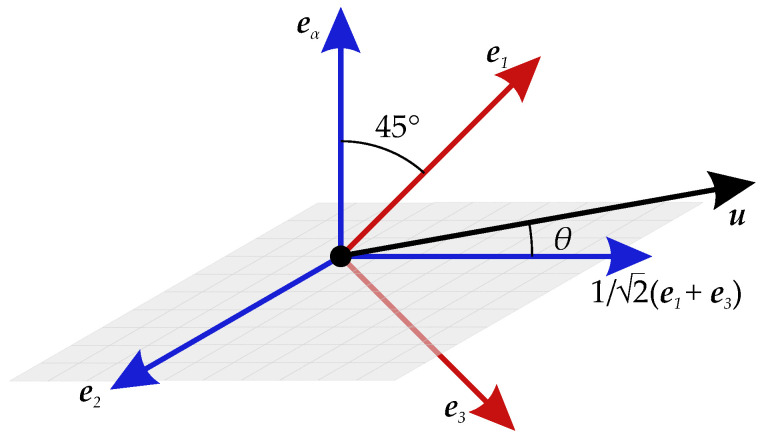
3D representation of the unit basis vectors. The unit basis vectors $e_{1}$, $e_{2}$, $e_{3}$ are the eigenvectors of the viscous stress tensor τ and correspond to maximum, intermediate, and minimum eigenvalues, respectively. The plane in gray is the shear plane. The unit basis vector $e_{\alpha }$ is normal to the shear plane. The angle θ is the angle between velocity vector u and $\frac{1}{\sqrt{2}}\left(e_{1}+e_{3}\right)$.

Note that also these unit vectors need only to be defined up to a sign, which does not matter in defining the symmetric conductivity tensor below Equation (6). Figure 4 shows a 3D representation of the unit basis vectors. In Figure 4, because the angle θ between u and $\frac{1}{\sqrt{2}}\left(e_{1}+e_{3}\right)$ is an acute angle, the first condition in Equations (1) is satisfied, hence the following:

(2) $$e_{\alpha }^{\mathrm{EV}}:=\frac{1}{\sqrt{2}}\left(e_{1}-e_{3}\right)\;.$$

#### 2.1.2. Velocity–Vorticity  Model

In the velocity–vorticity model, the unit basis vector $e_{\alpha }$, which defines the direction of the symmetry axis of the RBC, is computed by the normalized cross product of velocity u and vorticity ω vectors as follows:

(3) $$e_{\alpha }^{\mathrm{VV}}=\frac{u\times \omega }{\left|u\times \omega \right|}\;,$$

where the superscript VV refers to the velocity–vorticity model.

The vorticity vector ω is defined as the curl of the velocity vector u as follows:

(4) ω=∇×u.

### 2.2. Definition of the Conductivity Tensor

We begin this section by briefly recalling that in the case of materials with anisotropic electrical conductivity, this needs to be described by a conductivity tensor σ of second order. Ohm’s law, connecting the electrical current density J and the electrical field E then attains the form [29] as follows:

(5) J=σ·E,

where the central dot indicates matrix–vector multiplication. In the isotropic case, the conductivity tensor is a multiple of the unit matrix I, σ=σI, with the scalar conductivity σ. In general, the conductivity tensor is symmetric and therefore represented by a symmetric matrix in every coordinate system. The diagonal elements of this matrix connect the strength of the electrical current in the direction of the coordinate axes with the electrical field in the same direction. The non-diagonal elements are called in-plane conductivities and they account for electric currents induced perpendicular to the electrical field in one of the coordinate directions. Because of the symmetry of σ, there exists a local orthonormal coordinate system of eigendirections, with regard to which no in-plane conductivities occur, such that in this coordinate system the matrix has diagonal form. The diagonal entries with regard to this coordinate system are the eigenvalues of the matrix and are called the principal conductivities.

Both models introduced above define one distinguished eigendirection, $e_{\alpha }$, of the conductivity tensor and assume that the conductivity is isotropic in the plane perpendicular to this direction. The conductivity tensor may therefore be defined by two quantities, the principal conductivity in the direction of the short axis of the RBCs, $\sigma_{\alpha }$, and the transverse conductivity $\sigma_{\beta }$. Having specified $e_{\alpha }$, either by the eigenvector model or the velocity–vorticity model, the conductivity tensor is therefore defined as follows:

(6) $$\sigma =\sigma_{\alpha }\left(e_{\alpha }⊗e_{\alpha }\right)+\sigma_{\beta }\left(I-e_{\alpha }⊗e_{\alpha }\right)\;.$$

Note that $e_{\alpha }$ determines the dominating short axis direction, but depending on the shear stress we expect various degrees of alignment of the normals. At high shear stresses, the RBCs are expected to be strongly aligned, while at low shear stresses the orientations are mostly random. How these conductivities are derived from the local shear stress and shear rates is described in the next section.

### 2.3. Calculation of the Average Conductivities

The Maxwell–Fricke theory [13], with the formulation introduced by Hoetink et al. [7] and Gaw et al. [10], is adopted in this study for the calculation of components of the conductivity tensor.

The Maxwell–Fricke theory provides the anisotropic conductivity of a fluid suspension with completely aligned spheroidal particles based on the volume fraction and the aspect ratio λ=a/b of the particles. That is, the principal conductivities in the direction of the short axis $\sigma_{a}$ and in either of the long axes $\sigma_{b}$ are calculated as follows:

(7) $$\frac{\sigma_{a,b}\left(\lambda \right)}{\sigma_{\mathrm{pl}}}=\frac{1-H}{1+(C_{a,b}\left(\lambda \right)-1)H}\;,$$

where $\sigma_{\mathrm{pl}}$ is the conductivity of the blood plasma and *H* is the volume fraction of RBCs in the blood, i.e., the hematocrit value. See Table 1 for the values of model parameters. The orientation factors $C_{a}$ and $C_{b}$ depend on the aspect ratio λ via a function M(λ) through the following [10]:

(8) $$C_{a}\left(\lambda \right)=1/M\left(\lambda \right)\;,$$

and

(9) $$C_{b}\left(\lambda \right)=2/\left(2-M(\lambda )\right)\;.$$

The function M(λ) in turn is computed for a<b as follows [10]:

(10) $$M\left(\lambda \right)=\frac{\phi \left(\lambda \right)-\frac{1}{2}\sin\left(2\phi (\lambda )\right)}{\sin^{3}\phi \left(\lambda \right)}\cos\phi \left(\lambda \right)\;,$$

with

(11) $$\phi \left(\lambda \right)=\arccos\left(\lambda \right)=\arccos\left(\frac{a}{b}\right)\;.$$

**Table 1:** Constant parameters of the conductivity and blood rheology models.

Description	Symbol	Value	Units	References
Particle aspect ratio	λ	0.38	[-]	[7,10,20,30]
Conductivity of the blood plasma	$\sigma_{\mathrm{pl}}$	1.3	S/m ^−1^	[31]
Volume fraction of RBCs in the blood	*H*	45	%	[31]
Short particle semiaxis	*a*	$1.52\times 10^{-6}$	m	[31]
Long particle semiaxis	*b*	$4\times 10^{-6}$	m	[31]
Membrane shear modulus	μ	$10^{-5}$	$kg/s^{2}$	[7,32]
Orientation/Disorientation constant	*k*	1	$s^{-1/2}$	[31,33]
Dynamic viscosity of the blood plasma	$\eta_{\mathrm{pl}}$	$4.8\times 10^{-2}$	kg m^−1^s^−1^	[7,10,34]
Blood density	$\rho_{\mathrm{bl}}$	1060	$kg/m^{3}$	[35,36]

In the flowing blood, RBCs deform due to shear stresses [7], most notably in the tank treading motion based on the maximum shear stress $\tau_{\max}$. Given an undeformed aspect ratio λ in stationary blood, Hoetink et al. [7] derived the shear stress-dependent aspect ratio $\lambda_{d}$ of the deformed RBCs as follows:

(12) $$\lambda_{d}\left(\tau_{\max}\right)=\lambda {\left[1+\frac{\tau_{\max}b}{4\mu }\right]}^{-3}\;,$$

where μ denotes the membrane shear modulus of the RBCs, see Table 1.

Inserting the last relation in (7) yields the shear stress-dependent principal conductivities of blood with perfectly aligned RBCs as follows:

(13) $$\frac{\sigma_{a,b}\left(\tau_{\max}\right)}{\sigma_{\mathrm{pl}}}=\frac{1-H}{1+\left(C_{a,b}\left(\lambda_{d}\left(\tau_{\max}\right)\right)-1\right)H}\;.$$

However, full alignment of the RBCs is only expected at very high shear stresses, while in the absence of shear stresses, the orientation of the RBCs minor axis is supposed to be random. The conductivity model will thus also need an interpolation between these two extreme cases based on the fraction of aligned RBCs. A model for this interpolation based on the maximum shear rate ${\dot{\gamma }}_{\max}$ is available from Gaw et al. [10]. The authors assume the conductivity of blood with randomly oriented RBCs is obtained from Equation (13) by substituting $C_{a,b}$ by the average orientation factor as follows:

(14) $$C_{r}=1/3\left(C_{a}+2C_{b}\right)\;.$$

**Figure 5:**
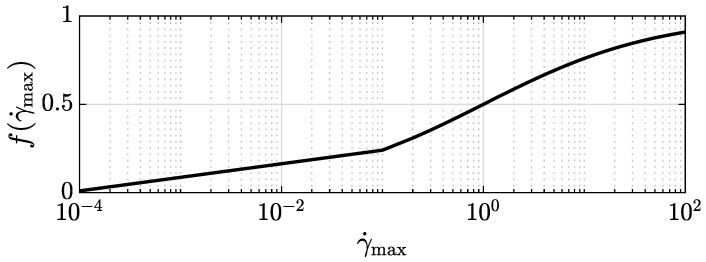
The fraction of aligned RBCs, computed by $f({\dot{\gamma }}_{\max})$, versus the maximum shear rate ${\dot{\gamma }}_{\max}$.

The interpolation of the conductivities between the fully aligned and the randomly oriented case is performed by interpolating the orientation factors based on the fraction of aligned RBCs given as a function of the maximum shear rate by the following:

(15) $$f\left({\dot{\gamma }}_{\max}\right)=\frac{{\dot{\gamma }}_{\max}}{{\dot{\gamma }}_{\max}+k\sqrt{{\dot{\gamma }}_{\max}}}\;.$$

where *k* is a constant whose value is indicated in Table 1. Function $f({\dot{\gamma }}_{\max})$ is plotted in Figure 5. Since this function provides a gradual transition from no alignment at vanishing shear rate to full alignment at an infinite shear rate, it does not provide a clear cut distinction as to what are high, intermediate, and low shear rates with regard to alignment. We suggest considering a fraction of aligned RBCs below 20% as low, beyond 80% as high, and as intermediate in-between. This defines shear rates to be considered low below a value of 0.0625 s^−1^, as high beyond 16 s^−1^, and as intermediate in-between.

The interpolated orientation factors $C_{\alpha }$ and $C_{\beta }$ are then defined as follows:

(16) $$C_{\alpha ,\beta }\left({\dot{\gamma }}_{\max},\tau_{\max}\right)=f\left({\dot{\gamma }}_{\max}\right)C_{a,b}\left(\tau_{\max}\right)+\left(1-f({\dot{\gamma }}_{\max})\right)C_{r}\left(\tau_{\max}\right)\;.$$

Eventually, the conductivities in the direction of the dominant alignment $e_{\alpha }$, $\sigma_{\alpha }$, and orthogonal to that $\sigma_{\beta }$ are computed as follows:

(17) $$\frac{\sigma_{\alpha ,\beta }\left({\dot{\gamma }}_{\max},\tau_{\max}\right)}{\sigma_{\mathrm{pl}}}=\frac{1-H}{1+(C_{\alpha ,\beta }\left({\dot{\gamma }}_{\max},\tau_{\max}\right)-1)H}\;.$$

Note that the shear rate is a unique function of shear stress and vice versa, such that the conductivity may as well be regarded as being either solely a function of the shear rate or of the shear stress. For low shear rates ${\dot{\gamma }}_{\max}<<1$, the aligned fraction becomes small, such that the two conductivities are approximately equal (and equal to the conductivity with randomly orientated RBCs) and the tensor is nearly isotropic. At high shear rates, when $\sigma_{\alpha }\approx \sigma_{a}$ and $\sigma_{\beta }\approx \sigma_{b}$ the anisotropy becomes maximal.

### 2.4. Computational Fluid Dynamics and Rheological Modeling

The Navier–Stokes equations model the blood flow as follow:

(18) $$\rho \left[\frac{\partial u}{\partial t}+\left(u\;\cdot \;\nabla \right)u\right]=-\nabla p+\nabla \;\cdot \;\tau \;,$$

with pressure *p* and the viscous stress tensor τ. The blood is modeled as an incompressible fluid and a constant density ρ; therefore, the mass balance equation reduces to ∇·u=0. Though blood is known to show shear thinning and a kind of flow stress [37], for large blood vessels it is admissible to model blood as a Newtonian fluid, resulting in a linear relation between the extra stress tensor τ and the rate-of-deformation tensor D.

(19) $$\tau =2\eta_{\mathrm{bl}}D\;.$$

The rate-of-deformation tensor D is defined as the symmetric part of the velocity gradient ∇u and is therefore computed as follows:

(20) $$D=\frac{1}{2}\left(\nabla u+\nabla u^{T}\right)\;.$$

The dynamic viscosity of blood $\eta_{\mathrm{bl}}$ is defined as a function of the hematocrit *H* introduced in Merrill [34], which reads as follows:

(21) $$\eta_{\mathrm{bl}}=\eta_{\mathrm{pl}}\left(1+2.5H+7.32H^{2}\right)\;.$$

Here, $\eta_{\mathrm{pl}}$ is the dynamic viscosity of the blood plasma. The kinematic viscosity of blood can then be computed as follows:

(22) $$\nu_{\mathrm{bl}}=\frac{\eta_{\mathrm{bl}}}{\rho_{\mathrm{bl}}}\;,$$

where $\rho_{\mathrm{bl}}$ is the density of blood. The values of $\eta_{\mathrm{pl}}$ and $\rho_{\mathrm{bl}}$ are constant, and their values are listed in Table 1.

The Newtonian modeling of blood as a suspension of cells and of the plasma as the carrier liquid of the cells means that well-known non-Newtonian rheological properties of blood are not accounted for. Non-Newtonian fluid behavior includes shear thinning, thixotropy, and viscoelasticity, which are seen in rheological material data of blood [38]. However, since electrical impedance changes due to blood flow variations can only be observed and measured for large vessels in which the effects of non-Newtonian behavior are small, the limitations imposed by the simplified model are acceptable.

### 2.5. Numerics

For CFD simulations of blood flow, the open-source CFD software OpenFOAM [39] is used. A new CFD solver is developed in OpenFOAM [39] to incorporate the conductivity models presented in Section 2.1.1, Section 2.1.2 and Section 2.3. The implementation of all the equations is performed for a general 3D flow.

OpenFOAM provides a spectral analysis of the viscous stress tensor τ, yielding the principal stresses (eigenvalues) $\tau_{1}>\tau_{2}>\tau_{3}$ and the corresponding eigendirections $e_{1}$, $e_{2}$, and $e_{3}$, respectively. The maximum shear stress is given by $\tau_{\max}=\tau_{1}-\tau_{3}$ and the maximum shear rate is obtained from the (isotropic) constitutive law as ${\dot{\gamma }}_{\max}=1/\left(2\eta_{\mathrm{bl}}\right)\tau_{\max}$.

## 3. Results

In this section, two CFD simulations are performed from which the electrical conductivity of blood with a hematocrit value of H=45% are calculated with the two suggested models. The first case is a fully developed steady-state laminar flow in a straight rigid vessel, while the second case regards the flow in a simplified aorta model, containing a rigid curved pipe imitating the aortic arch.

The first case is motivated by the extensive research and comprehensive understanding of blood conductivity within a straight rigid vessel, as reported in previous studies by Hoetink et al. [7], Gaw et al. [10]. This specific axisymmetric flow has distinctive characteristics. The shear planes are tangent to cylinders of constant radius, and, consequently, the normals of the shear planes point in radial direction. Moreover, besides the velocity vector pointing in axial direction, the vorticity vector points in circumferential direction in the cylindrical flow. Likewise, the cross-product of velocity and vorticity vector thus points in a radial direction, such that both models coincide in assuming the dominant short axis direction $e_{\alpha }$ in radial direction. For this simulation, the average inlet velocity $\bar{u}$ is 0.008 m s^−1^ and the vessel diameter *D* is 0.04 m. With a hematocrit *H* of 45%, the kinematic viscosity of blood $\nu_{\mathrm{bl}}$, according to Equations (21) and (22), has a value of $4.59\times 10^{-5}$ m^2^/s. The Reynolds number Re is computed as follows:

(23) $$Re=\frac{\bar{u}D}{\nu_{\mathrm{bl}}}=6.96\;.$$

**Figure 6:**
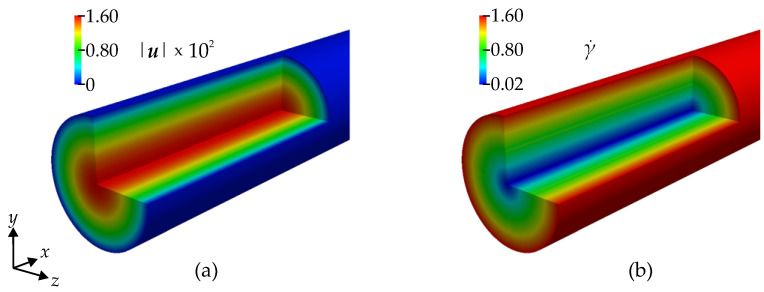
Color contour of (**a**) velocity magnitude $\left|u\right|\times 10^{2}$ in m s^−1^, and (**b**) shear rate $\dot{\gamma }$ in s^−1^.

**Figure 7:**
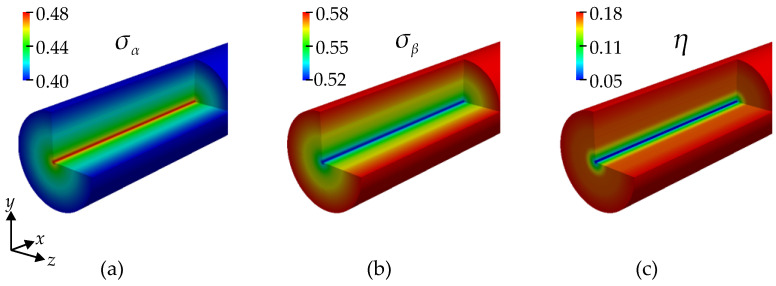
Color contours of (**a**) $\sigma_{\alpha }$ in S m^−1^, (**b**) $\sigma_{\beta }$ in S m^−1^, and (**c**) anisotropic indicator η.

Velocity and shear rate color contours in the cylindrical flow are illustrated in Figure 6. The velocity in the center is maximal, while at the wall it is zero due to the no-slip boundary condition. The shear rate is minimal at the vessel’s centerline and maximal near the wall. The shear stress follows the same pattern due to the linear isotropic constitutive law. In order to analyze the predicted conductivity tensor, we display the conductivities $\sigma_{\alpha }$ and $\sigma_{\beta }$ in Figure 7, which are, in the current case, the radial conductivity and, the conductivity in a tangential plane, i.e., equally, say, in the axial and circumferential direction, respectively. The conductivity $\sigma_{\beta }$ has its maximum near the walls, and $\sigma_{\alpha }$ is minimum there.

In order to quantify the anisotropy of the conductivity, we introduce an indicator η, which relates the maximal in-plane conductivity to the average conductivity, i.e., the difference between the largest and smallest principal conductivity over the sum of the principal conductivities, and is as follows:

(24) $$\eta =\frac{\sigma_{\beta }-\sigma_{\alpha }}{2\sigma_{\beta }+\sigma_{\alpha }}\;.$$

A contour plot of the anisotropy indicator is provided in the right-most picture in Figure 7. The conductivity is found to be nearly isotropic (low η) in the center of the cylinder but quickly reaches a high level away from the centerline. This is consistent with the expectation that the RBCs tend to have a more random orientation toward the center of the cylinder and are highly aligned in the higher shear stresses closer to the wall.

### Model Comparison

In the cylindrical flow treated model so far, the two suggested models coincide. In the current section, we regard a case where the models yield different conductivity tensors and quantify these differences. The current CFD simulation is a laminar steady-state blood flow in an idealized 3D aorta. This case is chosen because the models are not intrinsically identical in this case. The inlet average velocity $\bar{u}$ of the aorta is 0.022 m s^−1^, and the diameter *D* is 0.024 m. With a hematocrit *H* of 45%, the kinematic viscosity of blood $\nu_{\mathrm{bl}}$, according to Equations (21) and (22) is again $4.59\times 10^{-5}$ m^2^/s. The Reynolds number Re is computed as follows:

(25) $$Re=\frac{\bar{u}D}{\nu_{\mathrm{bl}}}=11.49\;.$$

**Figure 8:**
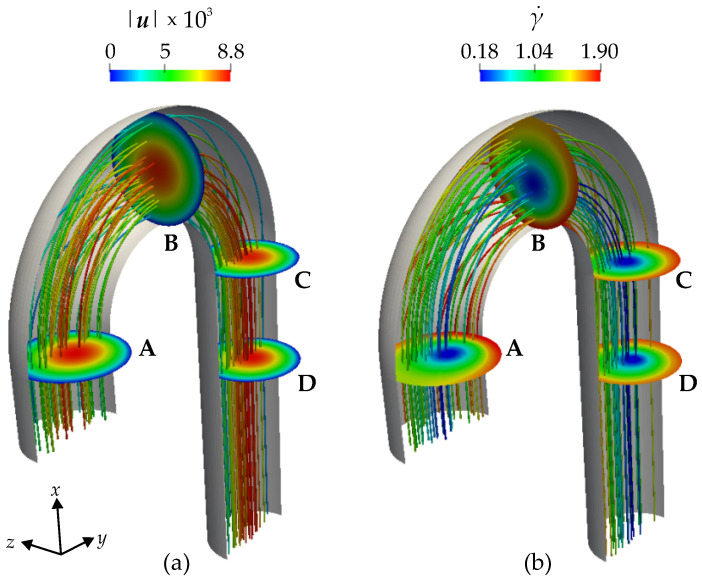
Color contours of (**a**) velocity magnitude $\left|u\right|\times 10^{3}$ in m s^−1^ and (**b**) shear rate $\dot{\gamma }$ in s^−1^. The color contours are illustrated in the cross-sections (A–D) and on the streamlines.

Figure 8 shows the color contours of the velocity magnitude |u| (a) in m s^−1^ and shear rate $\dot{\gamma }$ (b) in s^−1^. The velocity magnitude is zero at the wall due to the no-slip boundary condition at the wall, and it increases towards the center line of the aorta. The values of the shear rate $\dot{\gamma }$ close to the wall are higher than they are farther away from the wall. Also, in the vicinity of the inlet and the outlet of the aorta, the streamlines are analogous to a Poiseuille flow. However, in the arch itself, we observe that streamlines can move from the center towards the wall and vice versa due to the curvature of the geometry.

The difference between the calculated conductivity tensors originates from the determined orientation of the RBCs with the eigenvector (EV) and the velocity–vorticity (VV) models. Therefore, we compare how the models predict the orientation of RBCs, which is determined by $e_{\alpha }$, and then how different orientation predictions impact the computation of conductivity tensor.

To quantify the difference between the models we regard the angle $\psi_{\alpha }$ between $e_{\alpha }^{\mathrm{EV}}$ and $e_{\alpha }^{\mathrm{VV}}$ on the one hand, and the ‘angle’ $\psi_{\sigma }$ between the tensors on the other hand.

The angle between the differently defined symmetry axes is obtained as follows:

(26) $$\psi_{\alpha }=\arccos\left(|\langle e_{\alpha }^{\mathrm{EV}},e_{\alpha }^{\mathrm{VV}}\rangle |\right)\;,$$

where 〈·,·〉 denotes the standard scalar product of two vectors.

To quantify the difference between the calculated conductivity tensors, the angle $\psi_{\sigma }$ between the tensors $\sigma^{\mathrm{EV}}$ and $\sigma^{\mathrm{VV}}$ is defined as follows:

(27) $$\psi_{\sigma }=\arccos\left(\frac{\left|\langle \sigma^{\mathrm{EV}},\sigma^{\mathrm{VV}}\rangle \right|}{∥\sigma^{\mathrm{EV}}∥\times ∥\sigma^{\mathrm{VV}}∥}\right)\;,$$

where 〈·,·〉 now denotes the induced standard scalar product of two tensors and ∥·∥ the induced tensor norm, i.e., the Frobenius norm of the matrix representing σ. Note that since $\sigma_{\alpha }$ and $\sigma_{\beta }$ are the same for both models, the norm of the tensors is the same as follows:

(28) $$∥\sigma^{\mathrm{EV}}∥=∥\sigma^{\mathrm{VV}}∥=\sqrt{\sigma_{\alpha }^{2}+2\sigma_{\beta }^{2}}\;,$$

such that no difference in ‘magnitude’ occurs (which would not be quantified by the angle $\psi_{\sigma }$). The two angles are actually related to each other by the following relation:

(29) $$\cos\psi_{\sigma }=\frac{1}{\sigma_{\alpha }^{2}+2\sigma_{\beta }^{2}}\left(\sigma_{\alpha }^{2}\cos^{2}\psi_{\alpha }+2\sigma_{\alpha }\sigma_{\beta }\left(1-\cos^{2}\psi_{\alpha }\right)+\sigma_{\beta }^{2}\left(1+\cos^{2}\psi_{\alpha }\right)\right)\;,$$

which may be derived by inserting the $e^{\mathrm{EV}}$ and $e^{\mathrm{VV}}$ in Equation (6) and forming the scalar product. With the ratio between the two principal conductivities, $\lambda_{\sigma }=\sigma_{\alpha }/\sigma_{\beta }$, this relation reads as follows:

(30) $$\cos\psi_{\sigma }=\frac{1}{\lambda_{\sigma }^{2}+2}\left(\lambda_{\sigma }^{2}\cos^{2}\psi_{\alpha }+2\lambda_{\sigma }\left(1-\cos^{2}\psi_{\alpha }\right)+\left(1+\cos^{2}\psi_{\alpha }\right)\right)\;.$$

From Equation (17), we see that the ratio $\lambda_{\sigma }$ is a function of the shear rate and a monotonously decreasing one at that. The minimal value attained in the current simulation with a maximal shear rate of about 1.9 (compare Figure 8) is therefore $\lambda_{\sigma ,\min}=\lambda_{\sigma }\left({\dot{\gamma }}_{\max}=1.9\right)\approx 0.77$, and the maximal angle $\psi_{\sigma }$ is obtained for the minimal ratio and the maximal angle between the symmetry axis of the two models, $\psi_{\alpha }=90^{\circ }$, which yields $\psi_{\sigma ,\max}\approx 11.6^{\circ }$.

**Figure 9:**
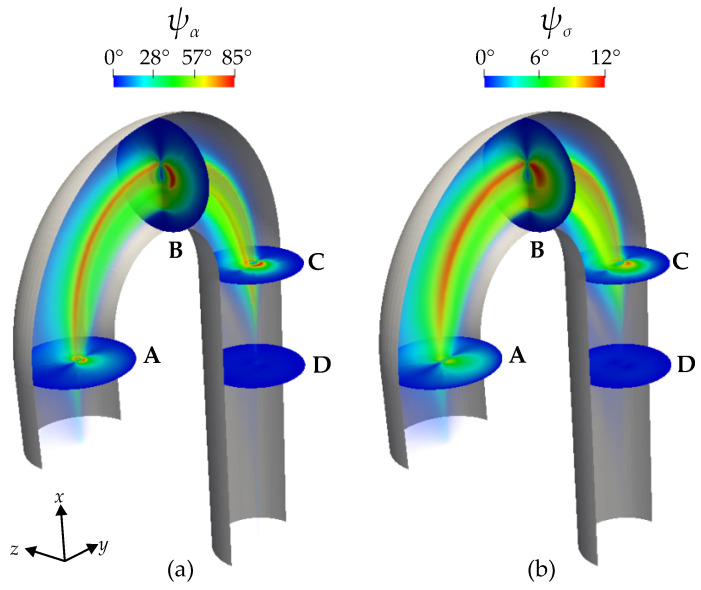
Color contours of (**a**) $\psi_{\alpha }$ in degrees and (**b**) $\psi_{\sigma }$ in degrees. The color contours are illustrated in the cross-sections (A–D) and in the volume of the aorta. $\psi_{\alpha }$ is the angle between $e_{\alpha }^{\mathrm{EV}}$ and $e_{\alpha }^{\mathrm{VV}}$ vectors resulting from the two models. $\psi_{\sigma }$ is the angle between the conductivity tensors $\sigma^{\mathrm{EV}}$ and $\sigma^{\mathrm{VV}}$ resulting from the two models.

Figure 9a displays the color contour of $\psi_{\alpha }$ in degrees. By definition, when $\psi_{\alpha }=0$, the two anisotropy models predict the same orientation of the RBCs and when $\psi_{\alpha }>0$, the models do not agree. In the proximity of the wall, where the viscous forces are dominant, $\psi_{\alpha }=0$, and therefore, the models are identical. In the arch, moving away from the immediate vicinity of the wall, it is evident that $\psi_{\alpha }>0$, reaching a peak at $\psi_{\alpha }\approx 85^{\circ }$. This is highlighted in the cross-sections A, B, and C. Cross-section A is at the beginning of the arch, B is in the middle, and C is at the end of the arch. The cross-sections (A–C) and the rainbow-like volume color contour show that the areas where the $\psi_{\alpha }>0$ expand from A to B and again shrink from B to C. Therefore, cross-section B highlights the maximum difference between the two models in predicting the orientation of the RBCs. At cross-section D, $\psi_{\alpha }=0$, a bit downstream of the arch, the flow is similar to a Poiseuille flow and the models agree.

Figure 9b, displays the color contours of $\psi_{\sigma }$ in degrees. The observed patterns closely resemble those of $\psi_{\alpha }$. This similarity is expected because the discrepancy in conductivity tensor computed in Equation (6) arises from the different $e_{\alpha }$ directions of the models, compare Equation (30). As already deduced from Equation (30) the values of $\psi_{\sigma }$ are much lower than those of $\psi_{\alpha }$ and only reach values of about $12^{\circ }$.

## 4. Discussion

The morphology of bioimpedance signals exhibits a high degree of dependence on the variations in the electrical conductivity of the blood flow. These conductivity variations are affected by the dynamic motion of RBCs, which are linked to the local hemodynamic conditions and disturbances of the flow. This suggests that an electrical conductivity model, capable of describing and incorporating local hemodynamic conditions, will eventually contribute to a better understanding of signal morphology and classification of the bioimpedance signals.

The key to understanding and classifying the influence of local pathophysiological flow disturbances on bioimpedance signals is to translate the microscopic effect of RBC motions into the macroscopic property of blood, i.e., anisotropic electrical conductivity. This study did just that by considering the macroscopic hemodynamic quantities, i.e., velocity, vorticity, shear rate, and shear stress. Two novel models, i.e., eigenvector and velocity–vorticity models, were developed to predict a spatially inhomogeneous and unsteady orientation and a deformation of RBCs and the anisotropic blood electrical conductivity. The two models are based on different assumptions for the dominating direction of the short axis of the RBCs. The principal conductivities of the anisotropic conductivity tensor are in both models, computed by adopting the blood-specific modifications of the Maxwell–Fricke theory.

The new models in this study overcome the limitations of previous 1D analytical formulations of Hoetink et al. [7] and Gaw et al. [10], and are a significant step towards modeling and understanding the electrical conductivity of blood. The models allow computing anisotropic blood conductivity as a field variable and, therefore, enable us to compute the conductivity through CFD simulations. Although the simulations performed in this study covered the particular case of laminar steady-state blood flow, the models are developed regardless of these assumptions.

The presented results are in good qualitative agreement with the analytical and experimental findings of Hoetink et al. [7], Gaw et al. [10], and Fischer et al. [21]. In the following, we shed light on the findings that are in agreement with the literature. In the straight rigid vessel flow, the changes in the velocity field only occur in the radial direction, and, therefore, shear rate and shear stress are only functions of the radial distance. In such a flow, the shear planes are tangent to cylinders of constant radius. Considering the analogies used in previous studies of Gaw et al. [10], Melito et al. [20], in the straight vessel flow, the larger principal value, $\sigma_{\beta }$, is the conductivity in the flow direction (and the circumferential direction), and $\sigma_{\alpha }$ is the conductivity in the radial direction. The simulation results of conductivity are in alignment with the statements of Hoetink et al. [7] and Gaw et al. [10] in the sense that in high shear rate zones, the conductivity in the flow direction is maximal, and the conductivity perpendicular to the flow direction is minimal.

The eigenvector and velocity–vorticity models are both identical for the simulation of blood flow in the straight rigid vessel; however, the simulation of blood flow in the idealized aorta showed that the models are not equivalent. The discrepancy in the symmetry axis predicted by the two models is mended by the limited anisotropy of the conductivity tensor. The anisotropy increases with shear stress, and since the highest angles between the symmetry axes occurred in the low shear stress (and thus low shear rate) areas, the difference between the models does not seem very strong. Further research is necessary to find out how the two models would differ in the predicted ICG signal one may obtain by inserting the conductivity tensor field from the CFD simulations in a 3D electric simulations, as was performed in [19]. ICG measurements at suitable simplified geometries could be used to validate the models and to determine which of the models yields better predictions. The validation of the models might also be performed via spatially resolved measurements, such as electrical impedance tomography [40].

On the modeling part, a key assumption was that the RBCs are oblate spheroids. Even though in the previous studies by Gaw et al. [10] such an assumption was also made and proved useful, we still suggest an investigation on the possibility of considering RBCs as triaxial ellipsoidal particles. The oblate spheroid assumption implies that in the principal coordinate system of RBCs, the principal conductivities in the direction of the long axes are equal. However, the experiments of Fischer et al. [21] and Minetti et al. [27] showed that the RBCs in tank-treading motion are triaxial ellipsoidal particles with a short, an intermediate, and a long axis. The latter suggests distinct conductivity values in the principal directions. In reality, red blood cells exhibit a biconcave morphology and, under various pathological conditions, the shape of RBCs may undergo further alterations. Further studies are required to investigate whether considering more details of the shapes improves the modeling of the electrical conductivity of blood. As a further simplification, this study assumed a constant value for the hematocrit. However, in the circuitry systems, the hematocrit may change, for instance, due to collision between RBCs and blood elements, the accumulation and adhesion of RBCs, and the bifurcation of blood vessels [41]. Considering that the presented models allow for the computation of the electrical conductivity of blood as a field variable, we suggest that future studies explore defining hematocrit as a field variable to account for multicellular collisions and compression.

It is important to acknowledge that the models presented in this study only account for the pathologies associated with geometric changes in the blood vessels. The pathologies that affect the shape of erythrocytes, such as sickle cell anemia or the electrochemical properties of blood, are not considered. These electrochemical properties, specifically the zeta potential of erythrocytes, play a key role in the repulsion of cells from one another [42]. A decrease in the zeta potential increases red blood cell aggregation at low shear rates, leading to a higher viscosity. A preliminary attempt to numerically model these effects has been presented in [43], and further investigations will examine their impact on viscosity and, subsequently, electrical conductivity.

Besides desisting from the shape and electrochemical details of the RBCs, we also simplified blood as a Newtonian fluid. However, blood is well-known for displaying non-Newtonian characteristics, like shear thinning, thixotropy, and viscoelasticity. Significant progress has been recently achieved in hemorheology, compare, for example, Giannokostas and Dimakopoulos [44], Giannokostas et al. [45], and Beris et al. [38]. The models developed in the present study can be seamlessly integrated with any blood rheology model since they only require input from hemodynamics without providing any feedback to the rheology. Although a simplified rheological model sufficed for evaluating the models, incorporating a suitable blood rheology model is recommended for model validation.

Furthermore, in our simulations, a rigid vessel wall was considered. However, when aiming at experimental validations, it might be necessary to consider the compliance of the vessel wall in a fluid–structure interaction model.

We finally note a slight discrepancy in our modeling, since the alignment of the RBCs, which effectuates an anisotropic electrical conductivity will likely also cause other material properties of blood to be anisotropic. Most notably, this would apply to the viscosity of blood, which would more consistently be modeled by a transversely isotropic second-order tensor. By contrast, we employ an isotropic constitutive law in Equation (19). Anisotropic hemodynamic models emerge, for example, from the conformation tensor used in modeling thixotropy [45]. The relation of the conformation tensor to the preferred orientation of the RBCs modeled in the current work will have to be explored in future work.

## 5. Conclusions

This study presents a novel approach to computing the electrical conductivity of blood. The new approach stems from determining the 3D inhomogeneous and unsteady motion of RBCs contributing to the anisotropic nature of the conductivity. In other words, this study established that variations in the anisotropic electrical conductivity of blood can be characterized by spatially inhomogeneous and unsteady changes in the orientation and deformation of RBCs. Two models were developed to compute conductivity as a tensor field variable. The CFD simulation of blood flow in the straight, rigid pipe showed that the computed conductivity tensor is consistent with experimental observations of Gaw et al. [10], Wtorek and Polinski [46], and Fischer et al. [21]. The CFD simulations of blood flow in the aorta showed that despite the differences between the models in the computations of the RBCs orientation, either of the models may be taken for the calculation of the conductivity tensor due to small discrepancies. While all the results are in qualitative agreement with the literature, validation of the models with experiments is yet outstanding.

The presented models for computing anisotropic conductivity allow computing the blood flow-related conductivity distribution in different locations in arteries; thus, the outcome might be very beneficial in investigating the possibility of using bioimpedance measurements as an alternative method for detecting CVDs and various pathologies, such as aortic stenosis, aneurysm, and aortic dissection. In addition, the findings of this study will potentially increase the accuracy of the simulation of bioimpedance signals by considering the effects of detailed blood flow.

## Data Availability

No new data were created or analyzed in this study. Data sharing is not applicable to this article.

## Abbreviations

The following abbreviations are used in this manuscript:

1D	One-dimensional
2D	Two-dimensional
3D	Three-dimensional
CFD	Computational fluid dynamics
CVD	Cardiovascular disease
CT	Computed tomography
ICG	Impedance cardiography
IPG	Impedance plethysmography
MRI	Magnetic resonance imaging
RBC	Red blood cell
